# Fatigue-Induced Changes in Knee-Extensor Mechanical Performance and Rectus Femoris Surface EMG During Repeated Isokinetic Knee Extensions

**DOI:** 10.3390/muscles5040072

**Published:** 2026-10-04

**Authors:** Smadar Peleg, Ophir Ravina, Eitan Shemy, Moshe Ayalon

**Affiliations:** Biomechanics Laboratory, Faculty of Sport and Movement Sciences, Levinsky-Wingate Academic College (Wingate Campus), Netanya 4290200, Israel

**Keywords:** performance fatigability, isokinetic dynamometry, knee extensors, surface electromyography, rectus femoris, peak torque, mean frequency, fatigue index

## Abstract

Performance fatigability is characterized by a decline in the capacity to maintain force or power during sustained or repeated activity. This exploratory study quantified knee-extensor mechanical performance and rectus femoris surface electromyographic (sEMG) changes during repeated maximal isokinetic knee extensions and explored sex-related differences. Twenty-nine healthy adults (20 men, 9 women; 20–31 years) performed 30 maximal concentric knee extensions at 180°·s^−1^. Mechanical outcomes were analyzed in all 29 participants, whereas rectus femoris sEMG outcomes were analyzed in 23 participants. Peak torque (PT) decreased by 32.75 ± 11.66% (adjusted *p* = 6.08 × 10^−11^), work per repetition by 33.35 ± 11.97% (adjusted *p* = 2.12 × 10^−11^), and mean frequency (MNF) by 23.44 ± 8.33% (adjusted *p* = 6.08 × 10^−11^). RMS increased significantly from Start to End (adjusted *p* = 0.000639), with a mean FI-RMS of −17.12 ± 23.11%. FI-PT and FI-work were very strongly associated (ρ = 0.938, adjusted *p* = 3.71 × 10^−13^), whereas no association involving rectus femoris MNF or RMS remained statistically significant after Holm–Bonferroni correction. No statistically significant sex-related differences were detected. Repeated maximal knee extensions reduced mechanical performance and rectus femoris MNF while increasing RMS. FI summarizes Start-to-End change across the measured outcomes; its reliability and validity require further investigation.

## 1. Introduction

Muscle fatigue is characterized by a progressive decline in the capacity of a muscle to produce force or power during sustained or repeated physical activity [1,2,3,4,5,6]. More specifically, performance fatigability refers to the objectively measurable decline in a performance outcome, such as force, power, or velocity, whereas perceived fatigue refers to the subjective sensation of tiredness or effort [7]. The mechanisms underlying muscle fatigue are multifactorial and involve changes at both central and peripheral levels of the neuromuscular system, including alterations in neural drive, neuromuscular transmission, muscle membrane excitability, action potential propagation, and metabolic processes [3,4,5,6]. Characterizing fatigue-related changes therefore requires consideration of both the mechanical decline in performance and accompanying changes in muscle electrical activity.

Several commonly used performance tests provide information regarding fatigability at the whole-body or multi-joint level. The Wingate anaerobic test, for example, quantifies peak power and the decline in power during a short, high-intensity cycling task and has traditionally been used to assess anaerobic performance and fatigability [8,9,10,11]. Vertical jump is also widely used to evaluate lower-limb explosive performance [12,13,14]. Although these tests provide useful information regarding overall physical performance, they do not isolate the response of a specific muscle group under controlled mechanical conditions. Isokinetic dynamometry enables muscle performance to be assessed at a predefined angular velocity and provides objective mechanical measures such as peak torque (PT) and work performed during individual repetitions [15,16]. Repeated maximal isokinetic contractions can therefore be used to characterize local muscle performance fatigability while controlling important features of the mechanical task.

Surface electromyography (sEMG) provides information regarding the electrical activity of a muscle during repeated contractions. Commonly examined sEMG variables include mean frequency (MNF), which characterizes the frequency distribution of the EMG power spectrum, and root mean square (RMS), which reflects the amplitude of the recorded EMG signal [17,18,19,20]. During fatiguing contractions, the sEMG power spectrum commonly shifts toward lower frequencies, resulting in a reduction in MNF [21,22]. In contrast, changes in RMS during dynamic fatiguing contractions are less consistent and may vary according to contraction characteristics and individual responses [21,22]. Together, MNF and RMS describe different frequency and amplitude-domain characteristics of the sEMG signal and, when interpreted alongside mechanical outcomes, provide different descriptive perspectives on fatigue-related responses during repeated maximal contractions.

Previous studies have characterized knee-extensor performance fatigability using repeated isokinetic contractions with different angular velocities, numbers of repetitions, and mechanical and electrophysiological outcomes [19,21,22,23,24,25,26,27,28,29,30,31,32]. Of particular relevance, Pincivero et al. [28] assessed quadriceps fatigability during 30 maximal concentric knee extensions at 180°·s^−1^, using both the ratio of work performed during the final five versus the initial five repetitions and the linear slope of work across all 30 repetitions. The present investigation extends this approach by concurrently examining two knee-extensor mechanical outcomes (PT and work per repetition) together with rectus femoris sEMG frequency- and amplitude-domain characteristics (MNF and RMS) during the same protocol, and by expressing their Start-to-End changes on a common percentage scale.

A further methodological consideration is how fatigue-related change is quantified. Previous studies have used several approaches, including comparisons of selected repetitions or predefined portions of repeated-contraction protocols and repetition-wise measures of performance decline [21,28,29,30,31,32,33]. A Fatigue Index (FI), based on the relative change between the beginning and end of a repeated-contraction protocol, provides a concise common framework for quantifying changes in mechanical and sEMG outcomes.

Sex-related differences in performance fatigability and neuromuscular responses have been reported, although their magnitude and direction vary according to muscle group, contraction mode, intensity, and experimental protocol [34,35,36]. Given the reported variability in sex-related fatigability responses, the present study also explored whether the magnitude of mechanical and sEMG changes during the same repeated-contraction protocol differed between women and men.

Therefore, the primary aim of this exploratory study was to quantify changes in knee-extensor mechanical performance between the beginning and end of 30 repeated maximal concentric knee extensions performed at 180°·s^−1^, using PT and work per repetition. The secondary aim was to characterize concurrent changes in rectus femoris sEMG using MNF and RMS. An additional exploratory aim was to examine whether the magnitude of these mechanical and sEMG changes differed between women and men.

We hypothesized that repeated maximal knee extensions would result in reductions in PT, work per repetition, and rectus femoris MNF, whereas changes in RMS would be less consistent. Given the exploratory nature of the sex comparison and the inconsistent findings reported previously, no directional hypothesis regarding sex differences was specified.

## 2. Results

### 2.1. Participants

Twenty-nine participants (20 men and 9 women) completed the study and were included in the mechanical analyses (Table 1). Peak torque (PT) and work per repetition (J·rep^−1^) were analyzed in all 29 participants. Usable rectus femoris sEMG data were available for 23 participants (14 men and 9 women); therefore, MNF and RMS analyses included these 23 participants. Six participants were excluded from the sEMG analyses because the rectus femoris channel was corrupted or absent during the fatigue set. No imputation was performed.

### 2.2. Start-to-End Changes in Mechanical Performance and Rectus Femoris sEMG

Substantial Start-to-End reductions were observed in both measures of knee-extensor mechanical performance. PT decreased from 126.02 ± 38.73 N·m at Start to 82.52 ± 23.39 N·m at End, corresponding to a mean decrease of 43.50 N·m (95% CI: 35.19–51.81) and an FI of 32.75 ± 11.66%. This change was statistically significant (t(28) = 10.72, d_z_ = 1.99, adjusted *p* = 6.08 × 10^−11^). Similarly, work per repetition decreased from 146.82 ± 43.12 J·rep^−1^ to 96.05 ± 28.31 J·rep^−1^, with a mean decrease of 50.77 J·rep^−1^ (95% CI: 41.62–59.92) and an FI of 33.35 ± 11.97% (t(28) = 11.37, d_z_ = 2.11, adjusted *p* = 2.12 × 10^−11^).

Rectus femoris MNF decreased from 94.87 ± 18.22 Hz at Start to 72.54 ± 15.93 Hz at End. The mean decrease was 22.33 Hz (95% CI: 18.56–26.11), corresponding to an FI of 23.44 ± 8.33% (t(22) = 12.27, d_z_ = 2.56, adjusted *p* = 6.08 × 10^−11^). RMS increased from 0.393 ± 0.161 mV at Start to 0.452 ± 0.188 mV at End. Because the paired RMS differences were non-normally distributed, this change was assessed using the Wilcoxon signed-rank test and was statistically significant (W = 32, matched rank-biserial r = −0.768, adjusted *p* = 0.000639). The median Start-to-End difference was −0.029 mV (95% bootstrap CI: −0.050 to −0.017), and the mean FI-RMS was −17.12 ± 23.11%. Thus, PT, work per repetition, and MNF decreased significantly, whereas RMS increased significantly from Start to End (Table 2).

### 2.3. Exploratory Sex-Related Differences

Exploratory comparisons showed no statistically significant differences between women and men in any of the four fatigue indices after Holm–Bonferroni correction.

For FI-PT, the median [IQR] was 28.49% [27.62–37.76] in women and 37.00% [28.65–40.87] in men (U = 60, r = −0.33, adjusted *p* = 0.555). The Hodges–Lehmann estimate of the female–male difference was −5.71 percentage points (95% CI: −16.96 to 1.30). For FI-J·rep^−1^, the corresponding values were 31.09% [30.64–42.19] in women and 34.78% [30.14–43.16] in men (U = 78, r = −0.13, adjusted *p* = 0.588), with a Hodges–Lehmann difference of −2.22 percentage points (95% CI: −13.67 to 6.19). No statistically detectable difference in FI-MNF was observed between women (18.35% [11.76–28.49]) and men (26.99% [20.88–31.29]; U = 39, r = −0.38, adjusted *p* = 0.555). The estimated female–male difference was −5.49 percentage points (95% CI: −13.63 to 1.47).

No statistically detectable sex-related difference was observed for FI-RMS. Women demonstrated a median FI-RMS of −14.56% [−19.80 to −11.71], compared with −3.89% [−34.03 to −0.61] in men. The between-sex comparison was not statistically significant (U = 41, r = −0.35, raw *p* = 0.176; Holm-adjusted *p* = 0.555), with a Hodges–Lehmann female–male difference of −9.90 percentage points (95% CI: −17.97 to 10.87) (Table 3).

### 2.4. Associations Among Fatigue Indices

A very strong positive association was observed between FI-PT and FI-J·rep^−1^ (Spearman’s ρ = 0.938, 95% CI: 0.843–0.969, adjusted *p* = 3.71 × 10^−13^). No other correlation remained statistically significant after Holm–Bonferroni correction. FI-PT was correlated with FI-MNF at ρ = 0.497 (95% CI: 0.066–0.777; adjusted *p* = 0.079) and with FI-RMS at ρ = 0.304 (95% CI: −0.150 to 0.699; adjusted *p* = 0.474). FI-J·rep^−1^ was correlated with FI-MNF at ρ = 0.422 (95% CI: 0.014–0.715; adjusted *p* = 0.180) and with FI-RMS at ρ = 0.227 (95% CI: −0.247 to 0.651; adjusted *p* = 0.564). FI-MNF and FI-RMS were correlated at ρ = 0.234 (95% CI: −0.235 to 0.607; adjusted *p* = 0.564) (Table 4).

### 2.5. Individual Fatigue Responses

**Figure 1:**
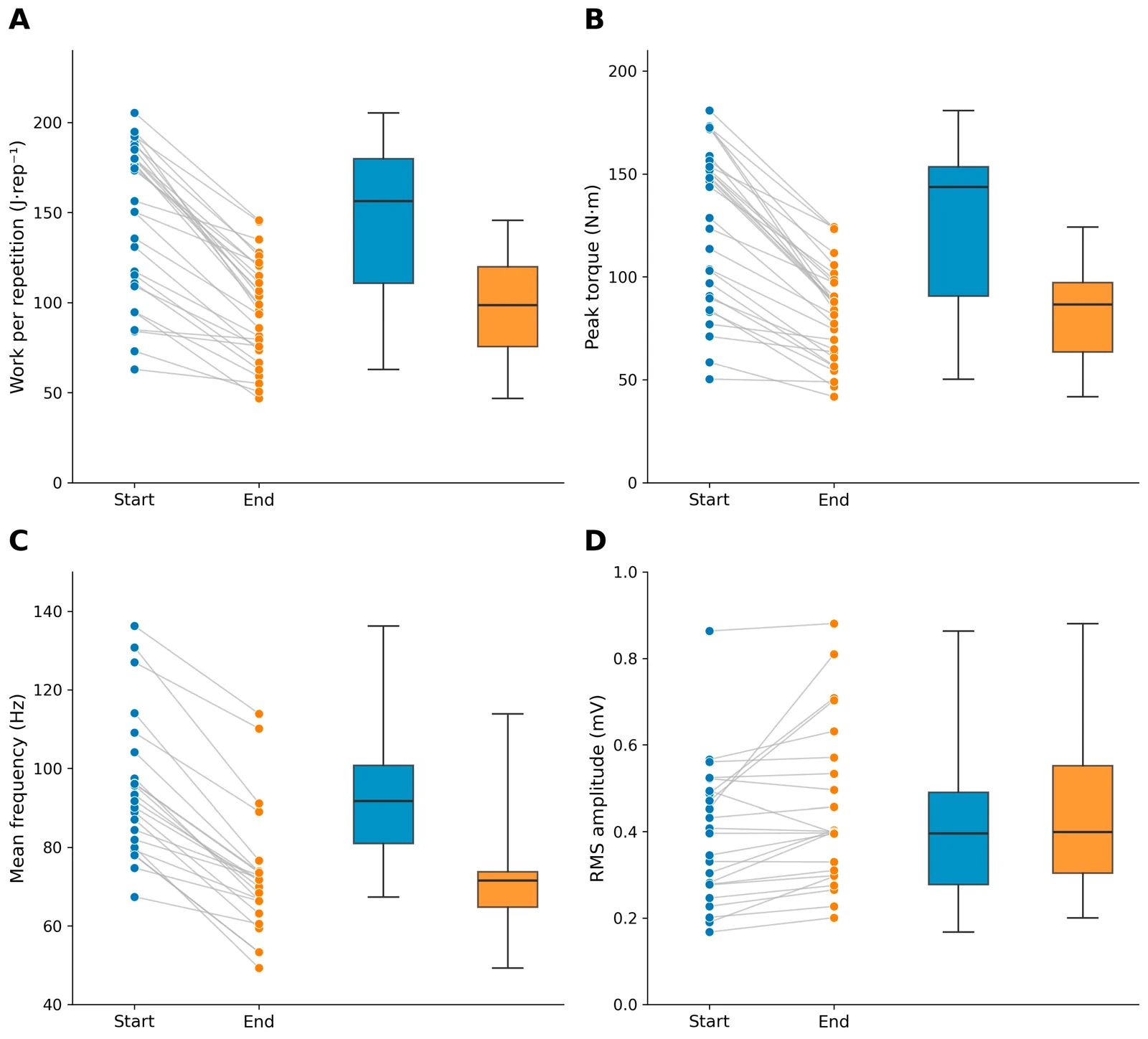
Individual Start-to-End changes in mechanical and rectus femoris sEMG outcomes. (**A**) work per repetition (J·rep^−1^), (**B**) peak torque (PT, N·m), (**C**) mean frequency (MNF, Hz), and (**D**) root mean square (RMS, mV). Blue—Start, Orange—End. Paired points represent individual participants; boxplots summarize group distributions, with whiskers indicating the observed range. Mechanical outcomes include 29 participants; sEMG outcomes include 23 participants.

Individual start-to-end changes in knee extensor mechanical performance and rectus femoris sEMG characteristics are illustrated in Figure 1. Most participants demonstrated reductions in PT, work per repetition, and MNF from Start (mean of repetitions 1–5) to End (mean of the final five complete repetitions), whereas RMS responses showed greater interindividual variability, with both increases and decreases observed. Individual paired observations illustrate the direction and magnitude of within-participant changes, while the accompanying boxplots summarize the group distributions at start and end. Importantly, Figure 1 represents only the predefined Start and End portions of the protocol and does not depict the progression of responses across all 30 repetitions.

## 3. Discussion

The present exploratory study examined changes in knee-extensor mechanical performance and rectus femoris surface electromyographic (sEMG) characteristics during 30 repeated maximal concentric knee extensions performed at 180°·s^−1^. The main findings were substantial Start-to-End reductions in peak torque (PT) and work per repetition, both of approximately 33%, accompanied by an approximately 23% reduction in rectus femoris mean frequency (MNF). Rectus femoris root mean square (RMS) increased significantly from Start to End, although considerable interindividual variability was evident. The fatigue indices for PT and work per repetition were very strongly associated, whereas no association involving rectus femoris MNF or RMS remained statistically significant after Holm–Bonferroni correction. No statistically significant sex-related differences were detected.

### 3.1. Changes in Knee Extensor Mechanical Performance

The approximately 33% reductions in both PT and work per repetition demonstrate a substantial decline in knee-extensor mechanical performance over the 30-repetition protocol. This finding is consistent with previous studies showing reductions in mechanical output during repeated maximal isokinetic knee-extension tasks [19,21,22,24,25,26,27,28,29,30,31,32]. Direct numerical comparisons across studies, however, should consider differences in angular velocity, number of repetitions, unilateral versus bilateral testing, range of motion, and the method used to quantify performance decline.

Of particular relevance, Pincivero et al. (2001) used a protocol closely comparable to that of the present study, consisting of 30 maximal concentric knee extensions performed at 180°·s^−1^ [28]. They quantified quadriceps fatigability using work performed during the final five relative to the initial five repetitions, as well as the linear slope of work across all 30 repetitions. The present protocol therefore shares the same number of maximal repetitions, contraction mode, angular velocity, and use of the initial and final five repetitions to characterize the change in mechanical performance. However, the present study expressed performance decline as the percentage reduction from Start to End and concurrently assessed PT and work per repetition together with rectus femoris sEMG characteristics. In contrast, Gerdle et al. examined 100 repeated maximal knee extensions at 90°·s^−1^, illustrating how differences in contraction velocity and protocol duration should be considered when comparing the magnitude of performance decline across studies [21].

PT and work per repetition decreased to a very similar extent in the present study (FI-PT: 32.75%; FI-J·rep^−1^: 33.35%), and their fatigue indices were very strongly correlated (ρ = 0.938). This close association is biomechanically expected because both variables are derived from torque production during the same knee-extension contractions. Strong associations between isokinetic peak torque and work have also been reported previously, indicating substantial overlap between these mechanical measures [37]. The present relationship should therefore be interpreted as evidence of substantial overlap rather than as independent confirmation of performance fatigability. Nevertheless, the two variables describe different features of mechanical performance: PT represents the highest instantaneous torque achieved during a repetition, whereas work reflects torque production across the angular displacement. Reporting both therefore provides different descriptive perspectives on the Start-to-End decline in knee-extensor mechanical performance.

### 3.2. Changes in Rectus Femoris sEMG Characteristics

Rectus femoris MNF decreased by approximately 23% from Start to End. A shift in the sEMG power spectrum toward lower frequencies during repeated fatiguing contractions has been widely reported and has also been demonstrated during repeated dynamic knee extensions [21,22]. Changes in spectral characteristics during fatigue may be influenced by several physiological processes, including alterations in muscle-fiber conduction velocity and the temporal characteristics of the recorded motor-unit action potentials [21,38]. The present reduction in MNF is most appropriately interpreted as a fatigue-related shift toward lower frequencies in the rectus femoris sEMG signal rather than as evidence of a specific neural mechanism [38].

RMS increased significantly from Start to End, resulting in a negative FI, although responses varied considerably among participants, with both increases and decreases evident in Figure 1. Previous work has similarly shown that sEMG amplitude during dynamic fatiguing contractions does not necessarily follow a uniform pattern [21,22,39]. RMS reflects the amplitude characteristics of the recorded sEMG signal and is influenced by multiple physiological and methodological factors [40,41]. Accordingly, the heterogeneous RMS responses observed here should not be interpreted directly in terms of increased or decreased motor-unit recruitment.

The present sEMG findings are based exclusively on recordings from the rectus femoris and should therefore be interpreted as reflecting the response of this muscle. Because only one muscle of the quadriceps group was recorded, the observed MNF and RMS responses cannot be generalized to the neuromuscular behavior of the quadriceps as a whole. Previous studies have demonstrated that the individual quadriceps muscles may exhibit different activation patterns during dynamic and fatiguing knee-extension tasks [42,43]. This distinction is particularly important when comparing the sEMG findings with PT and work per repetition, as these mechanical outcomes reflect the overall mechanical output of the knee extensors, including the contribution of all four heads of the quadriceps, whereas the sEMG measurements were obtained from the rectus femoris only.

### 3.3. Associations Between Mechanical and sEMG Changes

After Holm–Bonferroni correction, no association involving FI-MNF or FI-RMS remained statistically significant. Thus, the present data did not provide statistically significant evidence after correction that larger reductions in knee-extensor mechanical performance were consistently associated with larger changes in the measured rectus femoris sEMG characteristics.

These findings should not be interpreted as demonstrating physiological independence between mechanical and electrophysiological responses. Mechanical performance represents the net output of the knee-extensor system, whereas surface EMG recorded from a single muscle characterizes selected electrical features of that muscle. Furthermore, changes in MNF and RMS are influenced by several physiological and technical factors and cannot be mapped directly onto changes in torque or work [21,22,39]. The absence of significant correlations in the present sample therefore indicates only that no consistent participant-level association between the magnitude of these changes was detected.

The very strong relationship between FI-PT and FI-J·rep^−1^ should also be distinguished from the lack of mechanical-sEMG associations. PT and work are mechanically related outcomes obtained from the same contractions, and strong associations between these isokinetic measures have been reported previously [37]. In contrast, MNF and RMS describe frequency- and amplitude-domain characteristics of the rectus femoris sEMG signal, which are influenced by multiple physiological and methodological factors and should not be interpreted as direct equivalents of mechanical output [40]. These findings therefore support considering the mechanical and sEMG variables as different descriptive aspects of the response to repeated maximal contractions, without assuming that they represent independent physiological processes.

More broadly, the concurrent assessment of mechanical performance and sEMG can be considered within a multidimensional approach to neuromuscular function, in which different measurement modalities characterize distinct aspects of the response to exercise. Rossi et al. (2026), for example, demonstrated changes in segmental bioimpedance variables, including phase angle, resistance, and reactance, following eccentric plantar-flexor exercise performed to volitional failure, providing localized physiological information beyond mechanical performance measures alone [44]. Similarly, Thomas et al. [45] combined posturographic measures with sEMG during upright stance and handstanding, illustrating how functional and electrophysiological measures can provide different perspectives on task-specific neuromuscular behavior. Although these studies involved different muscle groups, contraction modalities, and functional tasks from the present isokinetic knee-extension protocol, they support the broader methodological value of multimodal assessment [45]. Future studies of performance fatigability could extend the present mechanical-sEMG approach by incorporating additional localized physiological or functional measures to provide a more comprehensive characterization of exercise-induced neuromuscular changes.

### 3.4. Exploratory Sex-Related Differences

No statistically significant sex-related differences were observed in FI-PT, FI-J·rep^−1^, FI-MNF, or FI-RMS after Holm–Bonferroni correction. The previous literature indicates that sex-related differences in performance fatigability are not uniform and may vary according to muscle group, contraction mode, intensity, and experimental protocol [35,36,46]. The present findings therefore do not support a general sex difference in the magnitude of knee-extensor performance decline under the conditions examined.

No statistically detectable sex-related difference was observed for FI-RMS. Women and men demonstrated median FI-RMS values of −14.56% and −3.89%, respectively (raw *p* = 0.176; Holm-adjusted *p* = 0.555). The Hodges–Lehmann estimate of the female–male difference was −9.90 percentage points (95% CI: −17.97 to 10.87). This finding should therefore not be interpreted as evidence of a sex-related difference in rectus femoris sEMG amplitude responses.

The small and unequal sex groups, particularly the nine women included in the analysis, limit the precision of these comparisons. In addition, factors that may influence sex-related fatigability, including training status and hormonal characteristics, were not systematically controlled. The present sex analyses are therefore best viewed as preliminary estimates that may inform the design and sample-size requirements of future studies specifically powered to examine sex-related differences.

### 3.5. Fatigue Index as a Summary of Performance Change

The FI provided a concise means of expressing the relative Start-to-End change in mechanical and sEMG outcomes on a common percentage scale. In the present study, the FI was calculated from the mean of the first five and final five repetitions. Averaging several repetitions at each end of the protocol reduces the influence of individual repetition-to-repetition variability compared with relying on single repetitions. A similar initial-to-final approach has previously been applied to repeated maximal knee-extension performance [28], although methods used to quantify fatigability vary considerably across studies [28,29,30,31,32,33].

The FI should nevertheless be interpreted specifically as a summary of Start-to-End change. Because the repetitions between the predefined Start and End portions are not represented in its calculation, the FI does not describe the repetition-by-repetition trajectory across the full protocol. Repetition-wise analyses, slopes, or other trajectory-based approaches can provide additional information regarding the rate and pattern of performance decline [28,30]. Thus, the FI and trajectory-based analyses address related but distinct aspects of performance fatigability.

The present findings establish the descriptive behavior of the FI within this protocol but do not establish it as a validated measurement instrument. Its test–retest reliability, measurement error, minimal detectable change, responsiveness, and validity remain to be determined. Accordingly, its current value is as a concise method for describing relative Start-to-End changes across outcomes rather than as a diagnostic or standardized measure of fatigability.

### 3.6. Study Limitations and Future Directions

Several limitations should be considered. First, the study included a relatively small convenience sample of healthy young adults, and no formal a priori sample-size calculation was performed. The study, and particularly the sex-related comparisons, should therefore be regarded as exploratory. This concern is especially relevant to the sEMG analyses, which included 23 participants after six recording exclusions, increasing uncertainty in the MNF and RMS estimates and in the corresponding sex comparisons.

Second, the FI compares predefined Start and End portions of the protocol and does not incorporate the repetition-by-repetition progression of performance between these portions. Interpretation of these changes should also consider the familiarization procedure. Although relatively extensive familiarization has been used in previous isokinetic studies to reduce learning-related variability and achieve stable performance [47], repeated familiarization contractions may themselves contribute to fatigue [48]. In the present study, participants completed 25 familiarization repetitions at progressively changing velocities and effort levels, followed by 2 min of recovery before the experimental protocol. Because recovery of torque or sEMG to a predefined baseline was not objectively verified, residual fatigue or potentiation cannot be excluded, particularly because repetitions 1–5 were used to define the Start value. Future studies should therefore examine repetition-by-repetition trajectories alongside Start-to-End indices, verify recovery following familiarization, and establish the reliability and measurement properties of the FI.

Third, sEMG was recorded only from the rectus femoris. The MNF and RMS findings therefore characterize this muscle and cannot represent the entire quadriceps muscle group. Because the Biodex and Delsys systems were not directly synchronized, the timing of individual sEMG contractions could not be verified against the mechanical active-extension phases. The sEMG windows were defined around detected peaks rather than confined to those phases and may have included varying portions of active extension and adjacent phases. This could affect MNF and RMS estimates and their associations with mechanical outcomes. Future studies should include additional knee-extensor muscles and synchronized sEMG and dynamometer recording.

Finally, factors potentially contributing to interindividual differences in fatigability, including habitual physical activity, training status, and sex-related physiological characteristics, were not systematically evaluated. Future adequately powered studies should examine these factors and determine whether the present findings extend to other age groups, athletic populations, and clinical populations.

### 3.7. Conclusions

Thirty repeated maximal concentric knee extensions at 180°·s^−1^ produced substantial reductions in knee-extensor mechanical performance, with PT and work per repetition decreasing by approximately 33%, accompanied by an approximately 23% reduction in rectus femoris MNF. Rectus femoris RMS increased significantly from Start to End, although considerable interindividual variability was evident.

The changes in PT and work per repetition were very strongly associated. No association involving rectus femoris MNF or RMS remained statistically significant after Holm–Bonferroni correction, and no statistically significant sex-related differences were detected.

The FI provided a concise description of the magnitude of Start-to-End change across the measured outcomes. However, it does not characterize the progression of fatigability across repetitions, and its measurement properties require further investigation before it can be considered a standardized measure of performance fatigability.

## 4. Methods

### 4.1. Participants and Study Design

This exploratory, within-subject laboratory study initially enrolled 31 healthy adults. Two participants (one man and one woman) were not included in the final dataset: one discontinued the protocol because of muscle discomfort, and one was excluded because the recorded data were unsuitable for subsequent processing. The final analytical sample therefore included 29 healthy adults (20 men and 9 women), aged 20–31 years (mean age: 25.1 ± 3.0 years).

Participants were recruited through WhatsApp groups of students at Levinsky-Wingate Academic College. Inclusion criteria were: (1) age 18–35 years; (2) no known musculoskeletal or neurological disorders affecting the lower extremities; and (3) no lower-limb injury during the 6 months preceding participation. Participants were excluded if they were unable to complete the testing protocol or experienced pain during testing.

No formal a priori sample size calculation was performed because the study was exploratory. Sex-related analyses were therefore considered exploratory. Physical activity level, training background, resistance training experience, and habitual sport participation were not systematically collected. Menstrual cycle phase, hormonal contraceptive use, and muscle mass were not collected or controlled.

The study was approved by the Institutional Review Board of Levinsky-Wingate Academic College (IRB No. 236, 2019) and was conducted in accordance with the Declaration of Helsinki. All participants received a detailed explanation of the study procedures and provided written informed consent before participation.

### 4.2. Study Protocol

#### 4.2.1. Anthropometric Measurements and General Warm-Up

Height was measured using a stadiometer (Tanita Leicester Height Measure, 2009, Leicester, UK), and body mass was measured using a digital scale (Life BL312, 2019, Beijin, China).

Prior to instrumentation and testing, participants completed a 5-min general warm-up consisting of treadmill jogging (Technogym, Cesena, Italy) at 8 km·h^−1^.

#### 4.2.2. Isokinetic Dynamometer Setup

**Figure 2:**
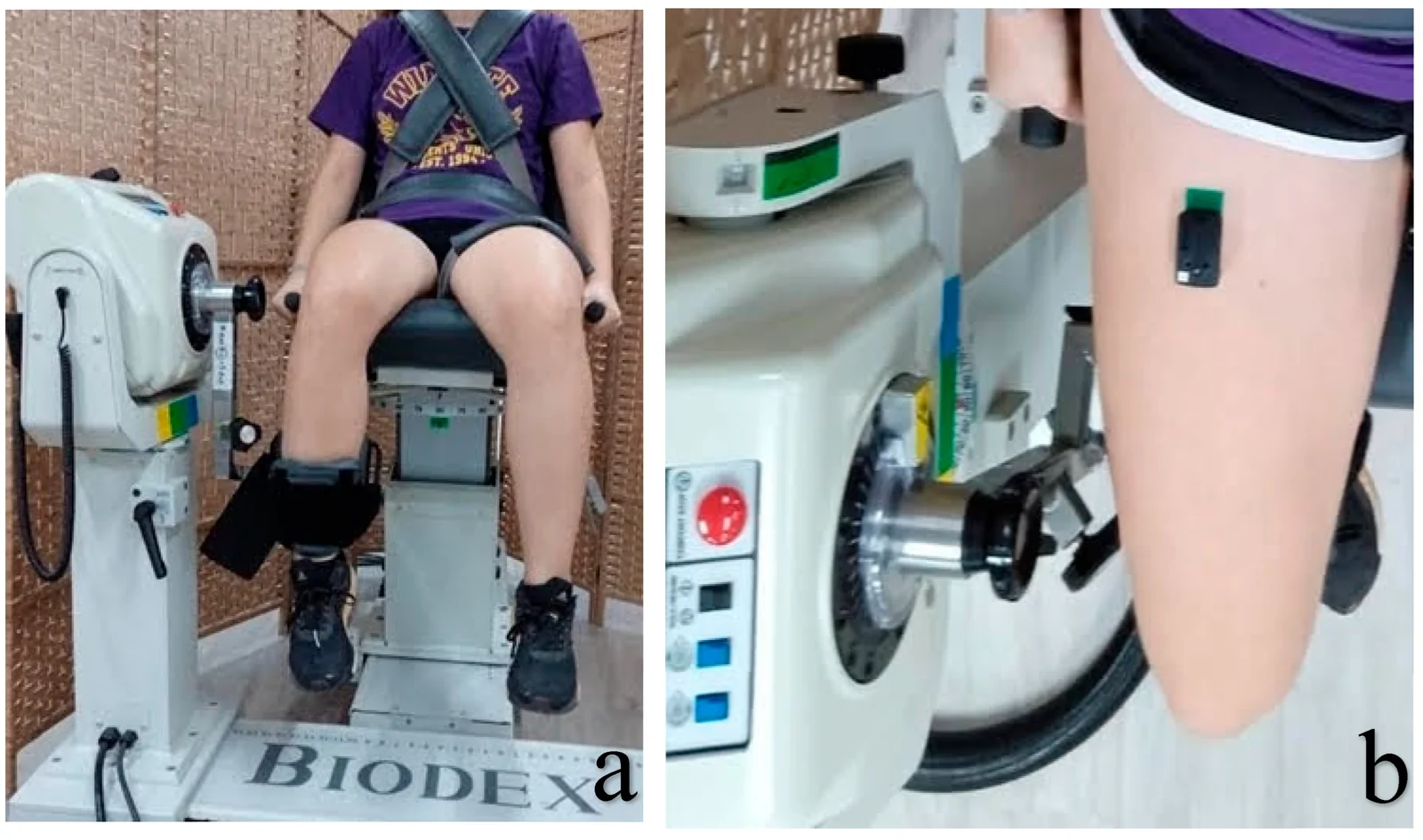
Experimental setup. (**a**) Participant positioning on the isokinetic dynamometer; (**b**) placement of the sEMG sensor on the rectus femoris.

Knee extension variables were assessed using an isokinetic dynamometer (Biodex System 4 Pro, Biodex Medical Systems, Shirley, NY, USA). The dominant lower limb was tested; dominance was defined as the limb the participant would preferentially use to kick a ball. Participants were seated and secured using the manufacturer’s stabilizing straps to minimize trunk and pelvic movement. The hip joint angle, defined as the angle between the trunk and thigh, was maintained at approximately 110° for all participants using a standardized backrest position. Seat position and lever arm length were individually adjusted according to participant anthropometry, while the anatomical knee axis was aligned with the dynamometer axis (Figure 2a). Approximately 1 cm of clearance was maintained between the proximal lower leg and the front edge of the seat, and the distal edge of the resistance pad was positioned approximately 1 cm proximal to the midpoint of the Achilles tendon. These individualized settings were not numerically recorded. The dynamometer was calibrated according to the manufacturer’s standard procedures before testing. Gravity correction was performed by weighing the relaxed leg lever system at 0° of knee flexion, and the resulting gravitational moment was accounted for in the recorded knee extension torque.

Torque and position data were exported at 100 Hz. No additional digital filtering was applied to the exported torque signal. Each active repetition was identified from the onset of knee extension movement to the reversal into the passive return phase, based on the direction of the lever arm velocity and the corresponding position data. All available complete repetitions were retained for analysis.

Testing was performed through a 95° range of knee motion, from 95 knee flexion to 0° (full extension). Accordingly, the dynamometer lever arm moved through a 95° range during each active concentric knee-extension phase.

#### 4.2.3. Fatigue Protocol

The experimental protocol consisted of 30 consecutive maximal concentric knee-extension contractions performed at an angular velocity of 180°·s^−1^. The protocol was designed to induce local knee-extensor performance fatigability while simultaneously recording mechanical and sEMG responses. Standardized verbal encouragement was provided throughout the 30 repetitions to promote maximal effort, while no visual performance feedback was provided.

Participants were instructed to exert maximal effort throughout the active knee-extension phase, during which the dynamometer moved through a 95° range of motion, from 95° of knee flexion toward full extension (0°). During the return phase, from 0° to 95° of knee flexion, participants were instructed to relax and allow the dynamometer to return the limb passively while minimizing voluntary muscle activation. The dynamometer cushioning was set at level 1, the lowest setting, thereby minimizing the acceleration and deceleration periods at the beginning and end of the active movement and maximizing the portion of the range of motion performed at the preset angular velocity. Thus, although brief acceleration and deceleration phases necessarily occurred, their contribution to the total movement was limited. Both the active knee-extension phase and the passive return phase were performed at 180°·s^−1^. Based on the exported Biodex position and velocity data sampled at 100 Hz, the median active-phase duration was 0.64 s (range: 0.59–0.69 s), the median passive-return duration was 0.67 s (range: 0.59–0.76 s), and the median complete-cycle duration was 1.31 s (range: 1.18–1.43 s). The median duration of 30 complete cycles was 39.35 s (range: 35.38–43.51 s). These durations include the brief acceleration and deceleration periods.

#### 4.2.4. Familiarization

Following the general warm-up and sEMG preparation, participants completed five familiarization sets of five concentric knee extensions at progressively decreasing angular velocities of 180, 150, 120, 90, and 60°·s^−1^. Effort was progressively increased across the first four sets without predefined percentage targets, and the final set was performed at approximately 90% of perceived maximal effort. A 30-s rest interval was provided between sets. Extensive familiarization protocols comprising 20 or more contractions have been used to reduce learning-related variability and establish stable performance [46,49]. After the fifth set, participants rested for 2 min before beginning the experimental fatigue protocol. No objective recovery criterion was used to determine this interval, and recovery of torque or sEMG to a predefined baseline was not verified.

#### 4.2.5. Surface Electromyography

Surface electromyographic (sEMG) activity was recorded from the rectus femoris of the tested limb using a Delsys Trigno Avanti wireless sensor (Delsys Inc., Natick, MA, USA) (Figure 2b). The sensor was positioned at 50% of the distance between the anterior superior iliac spine and the superior border of the patella and oriented longitudinally along this line, in accordance with SENIAM recommendations [50]. The sensor contained fixed parallel electrodes with an inter-electrode distance of 10 mm. Before placement, the site was shaved, lightly abraded, and cleaned with alcohol. The sensor was securely attached and remained in the same position throughout the session.

The sEMG signal was sampled at approximately 1926 Hz and hardware band-pass-filtered between 20 and 450 Hz. The Biodex and Delsys systems were not directly synchronized. Rectus femoris signals were therefore processed independently from the archived raw Delsys recordings. Repetitions were identified from a 0.200-s moving-RMS envelope of the median-centered rectus femoris signal. Peaks were detected at least 0.85 s apart. The prominence threshold was reduced stepwise through factors of 0.30, 0.22, 0.16, 0.11, 0.07, 0.04, 0.02, and 0 times the 75th percentile of the envelope until enough peaks were found. Among contiguous sequences of the required length, the sequence minimizing a penalty for irregular interpeak intervals was selected, with ties resolved by the highest mean log-envelope value at the peaks. The selected sequence was visually checked for each participant. No peak positions were manually adjusted.

MNF was calculated from the unrectified signal within a 0.625-s window centered on the detected sEMG peak after shifting the window center 0.075 s earlier. No additional tapering or overlap was applied. With the 0.625-s analysis window, the nominal frequency resolution was approximately 1.6 Hz. Spectral power was obtained using the real-valued fast Fourier transform (rFFT), and MNF was defined as the first spectral moment of the power spectrum:

$$MNF=\frac{\sum fP(f)}{\sum P(f)}$$

where f represents frequency and P(f) represents the corresponding spectral power.

RMS was calculated from a 0.400-s window centered on the same sEMG peak shifted 0.075 s earlier. The segment mean was removed before RMS was calculated as the square root of the mean squared signal. The 0.625-s MNF window provided the approximately 1.6-Hz nominal spacing reported above, whereas the 0.400-s RMS window measured amplitude over a shorter interval around the same sEMG peak. The fixed 0.075-s offset placed the center of both windows before that peak. Because these windows were defined by the sEMG peak rather than by the mechanical boundaries of active extension, they could include signal outside that phase, particularly when active extension lasted only 0.59 s. RMS was not normalized to an MVC or to the initial repetitions and was converted from volts to millivolts for reporting. Within each independently identified sEMG repetition sequence, contractions were numbered in temporal order. Repetitions 1–5 were assigned to the Start block and the final five to the End block, corresponding by repetition order to the mechanical Start and End blocks. For the two participants with 29 detected repetitions, the End block comprised repetitions 25–29; for all others, it comprised repetitions 26–30. Because the Biodex and Delsys systems were not directly synchronized, this correspondence was based on repetition order rather than direct temporal alignment.

#### 4.2.6. Data Analysis

To characterize the change from the beginning to the end of the fatigue protocol, start values were calculated as the mean of repetitions 1–5 and end values as the mean of the final five complete repetitions. In two participants with 29 complete repetitions, repetitions 25–29 were used for the End value; in all other participants, repetitions 26–30 were used. Averaging five repetitions was used to reduce the influence of individual repetition-to-repetition variability.

The mechanical outcomes were peak torque (PT, N·m) and work per repetition (J·rep^−1^)**,** representing knee-extensor mechanical performance. PT was defined as the maximum torque produced during the active concentric knee-extension phase. Raw torque, position, and velocity data were exported from the Biodex at 100 Hz. Work per repetition was calculated offline from the exported torque angle data during the active concentric extension phase using trapezoidal numerical integration after conversion of angular position to radians. Because anatomical knee angle decreased during extension, angular displacement was sign-corrected as θ_i_ − θ_i+1_, where θ_i_ and θ_i+1_ represent the knee angles at two consecutive measurement points, respectively, thereby yielding positive work values for concentric knee extension.

The sEMG outcomes were root mean square (RMS) and mean frequency (MNF, Hz)**,** representing amplitude- and frequency-domain characteristics, respectively, of the rectus femoris sEMG signal. Signal acquisition and processing procedures are described in the Surface Electromyography section.

To quantify the relative Start-to-End changes in each outcome, a Fatigue Index (FI, %) was calculated as:

FI (%) = [(X_Start_ − X_End_)/X_Start_] × 100.

where X_Start_ represents the mean of repetitions 1–5 and X_End_ represents the mean of the final five complete repetitions (repetitions 25–29 for the two participants with 29 complete repetitions and repetitions 26–30 for all others).

FI-MNF and FI-RMS represent relative Start-to-End changes in rectus femoris sEMG characteristics and should not be interpreted as validated indices of performance fatigability.

#### 4.2.7. Statistical Analysis

Final data processing and statistical analyses were performed in Python (version 3.12.14) using NumPy (version 2.3.5) and SciPy (version 1.17.0). Statistical significance was set at α = 0.05. Continuous variables are presented as mean ± standard deviation (SD) or median [interquartile range], as appropriate. For the Start-to-End analyses, normality of the paired differences was assessed using the Shapiro–Wilk test. The paired differences were consistent with normality for PT (W = 0.983, *p* = 0.916), work per repetition (W = 0.975, *p* = 0.708), and MNF (W = 0.972, *p* = 0.746), and these outcomes were analyzed using paired-samples *t*-tests. RMS differences were non-normally distributed (W = 0.823, *p* = 0.000916) and were therefore analyzed using the Wilcoxon signed-rank test. For the paired *t*-tests, effect size was expressed as Cohen’s dz and 95% CIs were calculated for the mean paired difference. For RMS, effect size was expressed as matched rank-biserial correlation and a bootstrap 95% CI was calculated for the median paired difference. Raw and Holm–Bonferroni-adjusted *p*-values are reported. The 95% CI was calculated using the t-distribution and the standard error of the paired differences. Cohen’s d_z_ was calculated by dividing the mean paired difference by the standard deviation of the paired differences.

Sex-related differences in the Fatigue Index (FI) were examined as exploratory analyses. Because of the small and unequal group sizes (9 women and 20 men), FI values were compared between women and men using the Mann–Whitney *U* test. Sex-specific FI values are presented as median [IQR]. Between-sex comparisons are reported using the Mann–Whitney *U* statistic, rank-biserial correlation as the effect-size estimate, and the Hodges–Lehmann estimate of the difference between women and men with its 95% CI.

Associations among the four fatigue indices (FI-PT, FI-J·rep^−1^, FI-MNF, and FI-RMS) were assessed using Spearman’s rank correlation coefficient (ρ), with 95% CIs. The bootstrap analyses used 20,000 resamples for each 95% confidence interval. For the RMS median paired difference, participants’ Start and End values were resampled together with replacement, and the median Start minus End difference was calculated in each resample. For the Hodges–Lehmann sex comparisons, women and men were resampled separately with replacement while preserving their respective group sizes; the median of all woman-minus-man differences was then calculated. For Spearman correlations, complete pairs of participant-level values were resampled with replacement, and ρ was recalculated. Confidence limits were defined by the 2.5th and 97.5th percentiles of the resulting bootstrap distributions.

To account for multiple comparisons, the Holm–Bonferroni procedure was applied separately to three predefined families of analyses: (1) the four Start-to-End comparisons (PT, J·rep^−1^, MNF, and RMS); (2) the four exploratory sex comparisons of FI values; and (3) the six pairwise correlations among the four fatigue indices. Adjusted *p*-values are reported, with an adjusted *p* < 0.05 considered statistically significant.

Analyses of peak torque (PT) and work per repetition (J·rep^−1^) included all 29 participants. Rectus femoris sEMG analyses of MNF and RMS included 23 participants (14 men and 9 women). Six participants were excluded from the sEMG analyses because the rectus femoris channel was corrupted or absent during the fatigue set. No imputation was performed.

## Figures and Tables

**Table 1:** Anthropometric and demographic characteristics of the final analytical sample, stratified by sex.

Variable	Women (*n* = 9)	Men (*n* = 20)	Total (*n* = 29)
Age (years)	24.9 ± 3.2	25.3 ± 3.0	25.1 ± 3.0
Height (cm)	161.2 ± 5.9	174.3 ± 8.3	170.3 ± 9.7
Body mass (kg)	56.7 ± 4.3	72.3 ± 10.6	67.4 ± 11.6
BMI (kg·m^−2^)	21.8 ± 1.3	23.7 ± 2.9	23.1 ± 2.6

Values are mean ± SD. BMI = body mass index.

**Table 2:** Start-to-End changes in mechanical and rectus femoris sEMG outcomes.

Outcome	Start	End	Δ (95% CI)	FI (%)	Statistics: Effect Size	*p* Raw; Adjusted
PT (N·m)	126.02 ± 38.73	82.52 ± 23.39	43.50 (35.19–51.81)	32.75 ± 11.66	t(28) = 10.72; d_z_ = 1.99	2.03 × 10^−11^; 6.08 × 10^−11^
Work (J·rep^−1^)	146.82 ± 43.12	96.05 ± 28.31	50.77 (41.62–59.92)	33.35 ± 11.97	t(28) = 11.37; d_z_ = 2.11	5.29 × 10^−12^; 2.12 × 10^−11^
MNF (Hz)	94.87 ± 18.22	72.54 ± 15.93	22.33 (18.56–26.11)	23.44 ± 8.33	t(22) = 12.27; d_z_ = 2.56	2.58 × 10^−11^; 6.08 × 10^−11^
RMS (mV)	0.393 ± 0.161	0.452 ± 0.188	−0.029 (−0.050 to −0.017)	−17.12 ± 23.11	W = 32; r_rb_ = −0.768	0.000639; 0.000639

Values are mean ± SD. PT = peak torque; Work = work per repetition; MNF = mean frequency; RMS = root mean square. Δ = Start-to-End difference (mean for PT, Work, and MNF; median for RMS), with 95% CI; FI = Fatigue Index. PT, Work, and MNF were analyzed using paired *t*-tests; RMS using Wilcoxon signed-rank. d_z_ = Cohen’s d_z_; W = Wilcoxon statistic; r_rb_ = matched rank-biserial correlation. Adjusted *p*-values are Holm–Bonferroni-corrected.

**Table 3:** Sex-related Start, End, and Fatigue Index values between women and men.

Outcome	Women, Median [IQR]	Men, Median [IQR]	FI Difference (95% CI)	U; r	*p* Raw; Adjusted
PT (N·m)	Start: 89.58 [71.04–97.04] End: 56.84 [54.50–63.54] FI (%): 28.49 [27.62–37.76]	Start: 150.89 [140.04–162.03] End: 89.34 [85.94–102.75] FI (%): 37.00 [28.65–40.87]	−5.71 (−16.96–1.30)	60; −0.33	0.164; 0.555
Work (J·rep^−1^)	Start: 109.18 [84.07–115.25] End: 66.63 [59.13–75.73] FI (%): 31.09 [30.64–42.19]	Start: 178.36 [154.89–187.54] End: 109.08 [97.86–123.13] FI (%): 34.78 [30.14–43.16]	−2.22 (−13.67–6.19)	78; −0.13	0.588; 0.588
MNF (Hz)	Start: 84.45 [78.05–96.07] End: 68.42 [66.41–72.87] FI (%): 18.35 [11.76–28.49]	Start: 92.52 [87.59–111.59] End: 71.66 [64.12–75.99] FI (%): 26.99 [20.88–31.29]	−5.49 (−13.63–1.47)	39; −0.38	0.139; 0.555
RMS (mV)	Start: 0.247 [0.202–0.305] End: 0.296 [0.265–0.396] FI (%): −14.56 [−19.80–−11.71]	Start: 0.480 [0.405–0.524] End: 0.515 [0.398–0.685] FI (%): −3.89 [−34.03–−0.61]	−9.90 (−17.97–10.87)	41; −0.35	0.176; 0.555

Women: *n* = 9; Men: *n* = 20 for PT and Work, and *n* = 14 for MNF and RMS. Values are median [IQR]. FI difference = Hodges–Lehmann estimate for women minus men. U = Mann–Whitney U statistic; r = rank-biserial correlation. Adjusted *p*-values are Holm–Bonferroni-corrected.

**Table 4:** Spearman correlations among the four Fatigue Indices.

Association	*n*	Spearman’s ρ	95% CI	Raw *p*	Adjusted *p*
FI-PT–FI-J·rep^−1^	29	0.938	0.843–0.969	6.18 × 10^−14^	3.71 × 10^−13^
FI-PT–FI-MNF	23	0.497	0.066–0.777	0.016	0.079
FI-PT–FI-RMS	23	0.304	−0.150–0.699	0.158	0.474
FI-J·rep^−1^–FI-MNF	23	0.422	0.014–0.715	0.045	0.180
FI-J·rep^−1^–FI-RMS	23	0.227	−0.247–0.651	0.297	0.564
FI-MNF–FI-RMS	23	0.234	−0.235–0.607	0.282	0.564

Raw *p*-values assess the significance of Spearman’s ρ; adjusted *p*-values were calculated using the Holm–Bonferroni procedure across the six correlations. Mechanical-only correlation included 29 participants; correlations involving sEMG outcomes included 23 participants.

## Data Availability

The datasets generated and/or analyzed during the current study are available from the corresponding author upon reasonable request.

## Abbreviations

FI, Fatigue Index; J·rep^−1^, joules per repetition; MNF, mean frequency; PT, peak torque; RMS, root mean square; sEMG, surface electromyography.
